# Consistent Recovery of Sensory Stimuli Encoded with MIMO Neural Circuits

**DOI:** 10.1155/2010/469658

**Published:** 2009-09-22

**Authors:** Aurel A. Lazar, Eftychios A. Pnevmatikakis

**Affiliations:** Department of Electrical Engineering, Columbia University, New York, NY 10027, USA

## Abstract

We consider the problem of reconstructing finite energy stimuli encoded with a population of spiking leaky integrate-and-fire neurons. The reconstructed signal satisfies a consistency condition: when passed through the same neuron, it triggers the same spike train as the original stimulus. The recovered stimulus has to also minimize a quadratic smoothness optimality criterion. We formulate the reconstruction as a spline interpolation problem for scalar as well as vector valued stimuli and show that the recovery has a unique solution. We provide explicit reconstruction algorithms for stimuli encoded with single as well as a population of integrate-and-fire neurons. We demonstrate how our reconstruction algorithms can be applied to stimuli encoded with ON-OFF neural circuits with feedback. Finally, we extend the formalism to multi-input multi-output neural circuits and demonstrate that vector-valued finite energy signals can be efficiently encoded by a neural population provided that its size is beyond a threshold value. Examples are given that demonstrate the potential applications of our methodology to systems neuroscience and neuromorphic engineering.

## 1. Introduction

Formal spiking neuron models, such as integrate-and-fire (IAF) neurons, encode information in the time domain [1]. Assuming that the input is bandlimited with a known bandwidth, a perfect recovery of the stimulus from the train of spikes is possible provided that the spike density is above the Nyquist rate [2]. Using results from frame theory [3] and statistics [4], these findings were extended to (i) bandlimited stimuli encoded with a population of IAF neurons with receptive fields modeled as linear filterbanks [5], (ii) multivariate (e.g., space-time) bandlimited stimuli encoded with a population of IAF neurons with Gabor spatiotemporal receptive fields [6], and (iii) sensory stimuli encoded with a population of leaky integrate-and-fire (LIF) neurons with random thresholds [7].

These results are based on the key insight that neural encoding of a stimulus with a population of LIF neurons is akin to taking a set of measurements on the stimulus. These measurements or encodings can be represented as projections (inner products) of the stimulus on a set of sampling functions. Stimulus recovery therefore calls for the reconstruction of the encoded stimuli from these inner products. These findings have shown that sensory information can be faithfully encoded into the spike trains of a neural ensemble and can serve as a theoretical basis for modeling of sensory systems (e.g., auditory, vision) [8].

In this paper we investigate the problem of reconstructing scalar and vector stimuli from a population of spike trains on a finite time horizon. The encoding circuits considered are either single-input multi-output or multi-input multi-output (MIMO). The increasing availability of multi-neuron population recordings has led to a paradigm shift towards population-centric approaches of neural coding and processing. Examples of MIMO models in systems neuroscience include extensive investigations of spike train transformations between neuron populations [9] as well as the analysis of the causal relationships between neurons in a population [10]. In neuromorphic engineering MIMO models have been used for brain-machine interfaces [11], as well as silicon retinas and related hardware applications [12].

The stimuli considered in this paper have finite energy and are defined on a finite time horizon. Even though restricted to finite time intervals, finite energy signals have infinite degrees of freedom. Consequently, the formal stimulus recovery is ill-defined. We cast the stimulus reconstruction problem in the abstract spline theory [13] and recover the stimulus as the unique solution to an interpolation spline problem. Splines serve as a valuable mathematical tool for interpolation problems, and their applications arise in many areas such as data smoothing in statistics [4], computer graphics [14], and digital signal processing [15].

Through the formulation of the interpolation spline problem, the reconstructed signal will give the same measurements as the original one. We show that this leads to a signal recovery that is* consistent* in the sense that the reconstructed signal triggers exactly the same spike train when passed through the same neuron as the original stimulus. The reconstructed signal is also required to achieve a maximum degree of smoothness gauged by a quadratic criterion. This condition ensures that the problem has a unique optimal solution.

A preliminary version of some of the ideas presented here appears in [16]. The analysis was based on results arising in generalized sampling [17]. Here the theory is presented in a more general setting using the spline theoretic framework, and all proofs are included. We apply our theoretical results to stimuli encoded with a number of spiking neural circuits of interest. These include populations of integrate-and-fire neurons with linear receptive fields that arise in hearing, ON-OFF neural circuits with feedback that arise in vision and multi-input multi-output (MIMO) neural circuits that arise in olfaction.

Formally, MIMO neural circuits encode *M*-dimensional vector-valued finite energy stimuli into the spike trains of a population of *N* neurons. Their architecture consists of an *N* × *M* linear, time invariant filtering kernel that feeds into an ensemble of *N* neurons. For this novel neural circuit we formulate and solve the problem of optimal consistent recovery and also discuss some of the key conditions that the filtering kernel has to satisfy in order to get a good reconstruction.

The paper is organized as follows. Section 2 formulates the problem of consistent reconstruction on a finite time horizon as a spline interpolation problem and presents its general solution. In Section 3 the reconstruction problem is addressed for stimuli encoded with a population of LIF neurons. Section 4 presents general MIMO neural encoding circuits and the corresponding optimal consistent stimulus reconstruction. A neuroscience inspired example is presented where the filtering kernel performs arbitrary (but known) delays and scalings to input stimuli akin to simple synaptic models. Finally Section 5 concludes our work.

## 2. Encoding with LIF Neurons and Consistent Stimulus Recovery

In this section we formulate and solve the problem of optimal consistent reconstruction for the simple case of a stimulus encoded with a single LIF neuron. We show how the spiking of an LIF neuron can be associated with a series of projections in the general *L* ^2^ space. We impose intuitive constraints on the desired reconstructed signal and show that the reconstruction algorithm can be reduced to a spline interpolation problem.

### 2.1. Neural Encoding with Single LIF Neurons

Let *u* = *u*(*t*), *t* ∈ [0, *T*], be a signal (or stimulus) of finite length and energy, that is, *u* ∈ *L* ^2^([0, *T*]). In what follows we assume that the stimulus *u* is the input to a Leaky Integrate-and-Fire (LIF) neuron. Throughout this paper (*t*_*k*_), *k* = 1, 2,…, *n*, denotes the set of recorded spikes. As in the case of bandlimited signals [5], neuron encoding can be associated with the projection (measurement) of the stimulus on a set of functions. By applying the *t*-transform [2], we can determine both the sampling functions and the projections of the stimulus on these functions using only the knowledge of the spike times.

Assume that the encoder is an LIF neuron, with threshold *δ*, capacitance *C*, resistance *R*, and constant bias *b*. The membrane potential of the LIF neuron is governed by the differential equation

(1) $$\begin{array}{c} C\frac{dV\left(t\right)}{dt}=-\frac{V\left(t\right)}{R}+u\left(t\right)+b \end{array}$$

with the initial condition *V*(0) = 0 and reseting conditions

(2) $$V\left(t_{k}\right)=\delta \Rightarrow \underset{t\to t_{k}^{+}}{\lim\;}V\left(t\right)=0$$

for all *t* ∈ [0, *T*], and *k* = 1, 2,…, *n*. By solving the differential equation in each interspike interval, the *t*-transform of the LIF neuron is given by

(3) $$\begin{array}{c} \int_{t_{k}}^{t_{k+1}}\left(u\left(s\right)+b\right)\exp\;\left(-\frac{t_{k+1}-s}{RC}\right)\;ds=C\delta \end{array}$$

for all *k*, *k* = 1, 2,…, *n* − 1. The *t*-transform can be rewritten as

(4) $$\begin{array}{c} L_{k}u=q_{k}, \end{array}$$

where *L*_*k*_ : *L* ^2^([0, *T*]) ↦ ℝ is a linear functional given by

(5) $$\begin{array}{c} L_{k}u=\int_{t_{k}}^{t_{k+1}}u\left(s\right)\exp\;\left(-\frac{t_{k+1}-s}{RC}\right)\;ds, \end{array}$$

(6) $$\begin{array}{c} q_{k}=C\delta -bRC\left(1-\exp\;\left(-\frac{t_{k+1}-t_{k}}{RC}\right)\right) \end{array}$$

for all *k* = 1, 2,…, *n* − 1. Therefore, we have the following result.

**Lemma 1**

*The *t*-transform of the LIF neuron can be written in inner-product form as*

(7) $$\begin{array}{c} \langle u,\phi_{k}\rangle =q_{k}, \end{array}$$

*where*

(8) $$\begin{array}{c} \phi_{k}\left(t\right)=\exp\;\left(-\frac{t_{k+1}-t}{RC}\right)1_{\left[t_{k},t_{k+1}\right]}\left(t\right), \end{array}$$

*and* 〈·, ·〉 : *L* ^2^([0, *T*]) × *L* ^2^([0, *T*]) ↦ ℝ *is the standard* *L* ^2^ *inner product restricted to the domain* [0, *T*] *for all* *k* = 1, 2,…, *n* − 1.

**Remark 2**

The inner products or projections 〈*u*, *ϕ*_*k*_〉, *k* = 1, 2,…, *n* − 1, in (7) represent a set of measurements or encodings of the signal *u* on [0, *T*]. Since (*ϕ*_*k*_) and (*q*_*k*_), *k* = 1, 2,…, *n* − 1, in (6) can be readily derived from the knowledge of the spike times and the neuron parameters, the signal encodings are available to an observer reading the spike times (*t*_*k*_), *k* = 1, 2,…, *n* − 1.

### 2.2. Consistent Stimulus Recovery

The problem of stimulus reconstruction calls for estimating the signal *u* given the set of spikes (*t*_*k*_), *k* = 1, 2,…, *n*. This problem is, for the class of stimuli *u* ∈ *L* ^2^([0, *T*]), ill-defined. (Signals that lie in a *L* ^2^ space have, in general, infinite degrees of freedom and thus cannot be perfectly recovered by a finite number of observations.) A remedy is provided by introducing a set of constraints on the recovery. The first constraint considered here requires the reconstructed signal $\hat{u}$ to generate the same spikes as the original stimulus. The second constraint requires choosing among the reconstructed stimuli the one with the maximum degree of smoothness. The latter is formulated as an optimization criterion.

**Definition 3**

A reconstruction $\hat{u}$ of *u* based on the spike times (*t*_*k*_), *k* = 1, 2,…, *n*, is said to be consistent if $\hat{u}$ triggers exactly the same spike train as the original stimulus *u*.

**Remark 4**

As before, assume that at time 0 the membrane potential of the LIF neuron is set to the resting potential 0. Then the consistency condition above is equivalent with

(9) $$\begin{array}{c} \langle u,\phi_{k}\rangle =\langle \hat{u},\phi_{k}\rangle \end{array}$$

for all *k*, *k* = 1, 2,…, *n* − 1.

**Definition 5**

A consistent reconstruction $\hat{u}$ that minimizes the quadratic criterion

(10) $$\left\|Ju\right\|={\left(\int_{0}^{T}{\left(\frac{d^{2}u}{ds^{2}}\right)}^{2}ds\right)}^{1/2}$$

is called the * optimal consistent reconstruction of u*.

**Remark 6**

‖*Ju*‖ is the norm of the second derivative of the reconstructed stimulus.

**Lemma 7**

*The optimal consistent reconstruction* $\hat{u}$ *solves the spline interpolation problem*

(11) $$\hat{u}=\underset{\langle u,\phi_{k}\rangle =q_{k}}{\text{argmin}}\left\|\frac{d^{2}u}{dt^{2}}\right\|,$$

*where* || · || *is the standard* *L* ^2^-*norm restricted to the interval* [0, *T*].

**Proof**

It follows directly from Definitions 3 and 5.

**Remark 8**

An introduction to splines and the general solution to spline interpolation problems is presented in the Appendix A.

**Theorem 9**

*The optimal consistent reconstruction is unique and is given by*

(12) $$\begin{array}{c} \hat{u}\left(t\right)=d_{0}+d_{1}t+\overset{n-1}{\underset{k=1}{\sum }}c_{k}\psi_{k}\left(t\right), \end{array}$$

*where*

(13) $$\begin{array}{c} \psi_{k}\left(t\right)=\left(\phi_{k}*{\left|\cdot \right|}^{3}\right)\left(t\right)=\int_{t_{k}}^{t_{k+1}}{\left|t-s\right|}^{3}\exp\;\left(-\frac{t_{k+1}-s}{RC}\right)\;ds, \end{array}$$

*where* ∗ *denotes the convolution, and* | · | *denotes the absolute value. With* **c** = [*c*_1_,*c*_2_,…,*c*_*n*−1_]^*T*^, **d** = [*d*_0_, *d*_1_] and **q** = [*q*_1_,*q*_2_,…,*q*_*n*−1_]^*T*^ *the coefficients* **c** *and* **d** *satisfy the matrix equations*

(14) $$\begin{array}{c} \left[\begin{array}{ccc} G & p & r\\ p^{T} & 0 & 0\\ r^{T} & 0 & 0 \end{array}\right]\left[\begin{array}{c} c\\ d_{0}\\ d_{1} \end{array}\right]=\left[\begin{array}{c} q\\ 0\\ 0 \end{array}\right]. \end{array}$$

*Moreover* **G** *is an* (*n* − 1) × (*n* − 1) *matrix, and* **p** *and* **r** *are column vectors with entries given by*

(15) $$\begin{array}{c} {\left[p\right]}_{k}=\langle \phi_{k},1\rangle ,\\ {\left[r\right]}_{k}=\langle \phi_{k},t\rangle ,\\ {\left[G\right]}_{kl}=\langle \phi_{k},\psi_{l}\rangle , \end{array}$$

*where all the inner products are restricted to the interval* [0, *T*].

**Proof**

The proof follows from Theorem 4 in Appendix A. Note that the function | · |^3^ is up to a multiplicative constant Green's function for the second-order iterated Laplacian. (See Lemma 5 in Appendix B).

The representation functions (13) can be explicitly given in analytical form as

(16) $$\begin{array}{ll} & \frac{\psi_{k}\left(t\right)}{{\left(RC\right)}^{4}}\\ & =\{\begin{array}{cc} f\left(\frac{t_{k+1}-t}{RC}\right)-f\left(\frac{t_{k}-t}{RC}\right) & \\ \;\times \exp\;\left(-\frac{t_{k+1}-t_{k}}{RC}\right), & t\leq t_{k},\\ 12\exp\;\left(-\frac{t_{k+1}-t}{RC}\right)+f\left(\frac{t_{k+1}-t}{RC}\right) & \\ \;+f\left(\frac{t_{k}-t}{RC}\right)\exp\;\left(-\frac{t_{k+1}-t_{k}}{RC}\right), & t_{k}<t\leq t_{k+1,}\\ f\left(\frac{t_{k}-t}{RC}\right)\exp\;\left(-\frac{t_{k+1}-t_{k}}{RC}\right) & \\ \;-f\left(\frac{t_{k+1}-t}{RC}\right), & t>t_{k+1}, \end{array} \end{array}$$

where *f*(*x*) = *x* ^3^ − 3*x* ^2^ + 6*x* − 6. The entries of the matrix **G** are given by

(17) $$\begin{array}{ll} & \frac{{\left[G\right]}_{kl}}{{\left(RC\right)}^{5}}\\ & =[g\left(\frac{t_{l+1}-t_{k+1}}{RC}\right)-g\left(\frac{t_{l+1}-t_{k}}{RC}\right)\exp\;\left(-\frac{t_{k+1}-t_{k}}{RC}\right)\\ & \;\;-g\left(\frac{t_{l}-t_{k+1}}{RC}\right)\exp\;\left(-\frac{t_{l+1}-t_{l}}{RC}\right)\\ & \;\;+g\left(\frac{t_{l}-t_{k}}{RC}\right)\exp\;\left(-\frac{t_{k+1}-t_{k}}{RC}-\frac{t_{l+1}-t_{l}}{RC}\right)]\cdot 1\left(k<l\right)\\ & \;+[6\left(1-\exp\;\left(-\frac{2\left(t_{k+1}-t_{k}\right)}{RC}\right)\right)\\ & \;\;\;-2g\left(\frac{t_{k+1}-t_{k}}{RC}\right)\exp\;\left(-\frac{t_{k+1}-t_{k}}{RC}\right)]\cdot 1\left(k=l\right)\\ & \;+[g\left(\frac{t_{k+1}-t_{l+1}}{RC}\right)-g\left(\frac{t_{k+1}-t_{l}}{RC}\right)\exp\;\left(-\frac{t_{l+1}-t_{l}}{RC}\right)\\ & \;\;\;-g\left(\frac{t_{k}-t_{l+1}}{RC}\right)\exp\;\left(-\frac{t_{k+1}-t_{k}}{RC}\right)\\ & \;\;\;+g\left(\frac{t_{k}-t_{l}}{RC}\right)\exp\;\left(-\frac{t_{l+1}-t_{l}}{RC}-\frac{t_{k+1}-t_{k}}{RC}\right)]\\ & \;\cdot 1\left(k>l\right) \end{array}$$

with *g*(*x*) = *x* ^3^ + 6*x*. Finally

(18) $$\begin{array}{ll} {\left[p\right]}_{k} & =RC\left(1-\exp\;\left(-\frac{t_{k+1}-t_{k}}{RC}\right)\right)\\ {\left[r\right]}_{k} & ={\left(RC\right)}^{2}\left(\left(\frac{t_{k+1}}{RC}-1\right)-\left(\frac{t_{k}}{RC}-1\right)\exp\;\left(-\frac{t_{k+1}-t_{k}}{RC}\right)\right). \end{array}$$

**Remark 10**

By letting *R* → ∞, one obtains the representation of the optimal consistent reconstruction for stimuli encoded with the ideal IAF neuron. The parameters and representation functions take a simple form:

(19) $$\begin{array}{cc} \underset{R\to \infty }{\lim\;}\phi_{k}\left(t\right) & =1_{\left[t_{k},t_{k+1}\right]}\left(t\right),\\ \underset{R\to \infty }{\lim\;}q_{k} & =C\delta -b\left(t_{k+1}-t_{k}\right),\\ \underset{R\to \infty }{\lim\;}\psi_{k}\left(t\right) & =\int_{t_{k}}^{t_{k+1}}{\left|t-s\right|}^{3}\;ds\\ & =\{\begin{array}{c} 0.25\left[{\left(t-t_{k+1}\right)}^{4}-{\left(t-t_{k}\right)}^{4}\right],\;t\leq t_{k},\\ 0.25[{\left(t-t_{k+1}\right)}^{4}\\ \;\;+{\left(t-t_{k}\right)}^{4}],\;t_{k}<t\leq t_{k+1},\\ 0.25\left[{\left(t-t_{k}\right)}^{4}-{\left(t-t_{k+1}\right)}^{4}\right],\;\;\;t>t_{k+1}, \end{array}\\ \underset{R\to \infty }{\lim\;}{\left[p\right]}_{k} & =t_{k+1}-t_{k},\\ \underset{R\to \infty }{\lim\;}{\left[r\right]}_{k} & =\frac{{\left(t_{k+1}\right)}^{2}-{\left(t_{k}\right)}^{2}}{2},\\ \underset{R\to \infty }{\lim\;}{\left[G\right]}_{kl} & =\{\begin{array}{cc} 0.05[{\left(t_{k+1}-t_{l+1}\right)}^{5}+{\left(t_{k}-t_{l}\right)}^{5} & \\ \;\;-{\left(t_{k}-t_{l+1}\right)}^{5}-{\left(t_{k+1}-t_{l}\right)}^{5}], & k<l,\\ 0.1{\left(t_{k+1}-t_{k}\right)}^{5}, & k=l,\\ 0.05[{\left(t_{l+1}-t_{k+1}\right)}^{5}+{\left(t_{l}-t_{k}\right)}^{5} & \\ \;\;-{\left(t_{l}-t_{k+1}\right)}^{5}-{\left(t_{l+1}-t_{k}\right)}^{5}], & k>l. \end{array} \end{array}$$

### 2.3. Example

**Figure 1:**
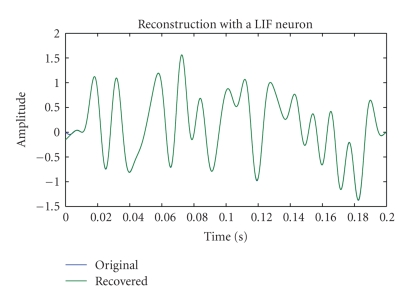
Encoding and reconstruction with a single LIF neuron.

The input to an LIF neuron is a bandlimited signal with bandwidth of 100 Hz. The neuron encodes the stimulus during the time interval [0, 0.2] second. A bias equal to *b* = 3 is also added to the input. The parameters of the LIF neuron are *δ* = 0.8, *C* = 0.01, and *R* = 50. Under these conditions the neuron generated 78 spikes. The recovered signal is shown in Figure 1. In order to quantify the quality of the recovery, we used the signal-to-noise ratio (SNR) defined by

(20) $$\begin{array}{c} \text{SNR}=10\;{\log\;}_{10}\left(\frac{{\left\|u\right\|}^{2}}{{\left\|u-\hat{u}\right\|}^{2}}\right). \end{array}$$

In the above SNR definition the noise corresponds to the error between the original and reconstructed signal. The SNR was equal to 47.53 dB.

## 3. Single-Input Multi-Output Encoding and Consistent Stimulus Recovery

In this section we consider the reconstruction of a stimulus encoded with a population of LIF neurons. We demonstrate that the consistent recovery can be again formulated as a spline interpolation problem and provide the reconstruction algorithm. We also show how the methodology developed in this section can be applied to a simple encoding circuit consisting of two-coupled ON-OFF neurons with feedback.

### 3.1. Encoding with a Population of LIF Neurons

In what follows we consider a neural encoding circuit consisting of *N* leaky integrate-and-fire (LIF) neurons. Neuron *j* has threshold *δ* ^*j*^, bias *b* ^*j*^, resistance *R* ^*j*^, and capacitance *C* ^*j*^ for all *j* = 1, 2,…, *N*. After each spike every neuron resets its membrane potential to 0. Let *t*_*k*_^*j*^ denote the *k*th spike of neuron *j*, with *k* = 1, 2,…, *n*_*j*_, where *n*_*j*_ is the number of spikes that the neuron *j* generates, *j* = 1, 2,…, *N*.

The *t*-transform of the population of *N* LIF neurons is given by

(21) $$\begin{array}{c} \int_{t_{k}^{j}}^{t_{k+1}^{j}}\left(u\left(s\right)+b^{j}\right)\exp\;\left(-\frac{t_{k+1}^{j}-s}{R^{j}C^{j}}\right)\;ds=C^{j}\delta^{j}. \end{array}$$

Let

(22) $$\begin{array}{c} q_{k}^{j}=C^{j}\delta^{j}-b^{j}R^{j}C^{j}\left(1-\exp\;\left(-\frac{t_{k+1}^{j}-t_{k}^{j}}{R^{j}C^{j}}\right)\right) \end{array}$$

for all *j* = 1, 2,…, *N*. As in the previous section, we have the following.

**Lemma 11**

*The *t*-transform of the LIF neuron can be written in inner-product form as*

(23) $$\begin{array}{c} \langle u,\phi_{k}^{j}\rangle =q_{k}^{j}, \end{array}$$

*where*

(24) $$\begin{array}{c} \phi_{k}^{j}\left(t\right)=\exp\;\left(-\frac{t_{k+1}^{j}-t}{R^{j}C^{j}}\right)1_{\left[t_{k}^{j},t_{k+1}^{j}\right]}\left(t\right), \end{array}$$

*and* 〈·, ·〉 : *L* ^2^([0, *T*]) × *L* ^2^([0, *T*]) ↦ ℝ *is the standard* *L* ^2^ *inner product restricted to the domain* [0, *T*] *for all* *k* = 1, 2,…, *n* *and* *j* = 1, 2,…, *N*.

### 3.2. Consistent Stimulus Recovery

Let **q** be a column vector defined as **q** = [**q** ^1^; **q** ^2^;…; **q** ^*N*^]^*T*^ with **q** ^*j*^ = [*q*_1_^*j*^, *q*_2_^*j*^,…, *q*_*n*_*j*_−1_^*j*^]^*T*^, *j* = 1, 2,…, *N*. The vectors **p**, **r**, **c** have the same dimension and are similarly defined. The matrix **G** is a block square matrix defined as

(25) $$\begin{array}{c} G=\left[\begin{array}{ccc} G^{11} & \cdots & G^{1N}\\ \vdots & \ddots & \vdots \\ G^{N1} & \cdots & G^{NN} \end{array}\right] \end{array}$$

with **G** ^*ij*^ ∈ ℝ^*n*_*i*_−1×*n*_*j*_−1^.

The following theorem first appeared in [16]; its proof is presented here for the first time.

**Theorem 12**

*Assume that at time 0 the membrane potential of all neurons is at the rest value 0. The optimal consistent reconstruction* $\hat{u}$ *is unique and can be written as*

(26) $$\begin{array}{c} \hat{u}\left(t\right)=d_{0}+d_{1}t+\overset{N}{\underset{j=1}{\sum }}\overset{n_{j}-1}{\underset{k=1}{\sum }}c_{k}^{j}\psi_{k}^{j}\left(t\right), \end{array}$$

*where*

(27) $$\begin{array}{c} \psi_{k}^{j}\left(t\right)=\left(\phi_{k}^{j}*{\left|\cdot \right|}^{3}\right)\left(t\right)=\int_{t_{k}^{j}}^{t_{k+1}^{j}}{\left|t-s\right|}^{3}\exp\;\left(-\frac{t_{k+1}^{j}-s}{R^{j}C^{j}}\right)ds. \end{array}$$

*The reconstruction coefficients are given in matrix form by*

(28) $$\begin{array}{c} \left[\begin{array}{c} c\\ d_{0}\\ d_{1} \end{array}\right]={\left[\begin{array}{ccc} G & p & r\\ p^{T} & 0 & 0\\ r^{T} & 0 & 0 \end{array}\right]}^{+}\cdot \left[\begin{array}{c} q\\ 0\\ 0 \end{array}\right], \end{array}$$

*where* [·]^+^ *denotes the pseudoinverse and*

(29) $$\begin{array}{ll} {\left[p^{j}\right]}_{k} & =R^{j}C^{j}\left(1-\exp\;\left(-\frac{t_{k+1}^{j}-t_{k}^{j}}{R^{j}C^{j}}\right)\right),\\ {\left[r^{j}\right]}_{k} & ={\left(R^{j}C^{j}\right)}^{2}(\left(\frac{t_{k+1}^{j}}{R^{j}C^{j}}-1\right)-\left(\frac{t_{k}^{j}}{R^{j}C^{j}}-1\right)\exp\;\left(-\frac{t_{k+1}^{j}-t_{k}^{j}}{R^{j}C^{j}}\right)),\\ {\left[G^{ij}\right]}_{kl} & =\langle \phi_{k}^{i},\psi_{l}^{j}\rangle . \end{array}$$

**Proof**

The proof is notationally more complex but closely follows the proof of Theorem 9. The representation functions (27) can be computed analytically as

(30) $$\begin{array}{ll} & \begin{array}{c} \frac{\psi_{k}^{j}\left(t\right)}{{\left(R^{j}C^{j}\right)}^{4}} \end{array}\\ & =\{\begin{array}{cc} f\left(\frac{t_{k+1}^{j}-t}{R^{j}C^{j}}\right)-f\left(\frac{t_{k}^{j}-t}{R^{j}C^{j}}\right) & \\ \;\times \exp\;\left(-\frac{t_{k+1}^{j}-t_{k}^{j}}{R^{j}C^{j}}\right), & t\leq t_{k}^{j},\\ 12\exp\;\left(-\frac{t_{k+1}^{j}-t}{R^{j}C^{j}}\right)+f\left(\frac{t_{k+1}^{j}-t}{R^{j}C^{j}}\right) & \\ \;+f\left(\frac{t_{k}^{j}-t}{R^{j}C^{j}}\right)\exp\;\left(-\frac{t_{k+1}^{j}-t_{k}^{j}}{R^{j}C^{j}}\right), & t_{k}^{j}<t\leq t_{k+1}^{j},\\ f\left(\frac{t_{k}^{j}-t}{R^{j}C^{j}}\right)\exp\;\left(-\frac{t_{k+1}^{j}-t_{k}^{j}}{R^{j}C^{j}}\right) & \\ \;-f\left(\frac{t_{k+1}^{j}-t}{R^{j}C^{j}}\right), & t>t_{k+1}^{j}, \end{array} \end{array}$$

where *f*(*x*) = *x* ^3^ − 3*x* ^2^ + 6*x* − 6.

### 3.3. Example: Encoding with an ON-OFF Neuron Pair

**Figure 2:**
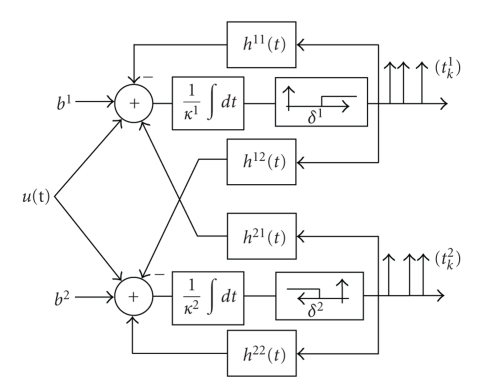
Coupled ON-OFF integrate-and-fire neurons.

We consider an encoding circuit consisting of two interconnected integrate-and-fire neurons with feedback. For simplicity we assume that the IAF neurons are ideal, that is, *R* ^1^, *R* ^2^ → ∞. Figure 2 depicts the circuit. Whenever a spike is generated, the firing neuron is reset and feedback is added to the membrane potential. In addition, the firing of each spike is communicated to the other neuron through cross-feedback. The two neurons in Figure 2 arise as models of ON and OFF bipolar cells in the retina and their connections through the nonspiking horizontal cells [18].

Let *t*_*k*_^*j*^ denote the *k*th spike of the *j*th neuron, *k* = 1,…, *n*_*j*_ and *j* = 1, 2. The *t*-transform of the neural circuit amounts to

(31) $$\begin{array}{ll} \int_{t_{k}^{1}}^{t_{k+1}^{1}}u\left(s\right)\;ds & =\kappa^{1}\delta^{1}-b^{1}\left(t_{k+1}^{1}-t_{k}^{1}\right)+\underset{l\leq k}{\sum }\int_{t_{k}^{1}}^{t_{k+1}^{1}}h^{11}\left(s-t_{l}^{1}\right)\;ds\\ & \;-\underset{l}{\sum }\int_{t_{k}^{1}}^{t_{k+1}^{1}}h^{21}\left(s-t_{l}^{2}\right)\;ds1_{\left\{t_{l}^{2}<t_{k}^{1}\right\},}\\ \int_{t_{k}^{2}}^{t_{k+1}^{2}}u\left(s\right)\;ds & =\kappa^{2}\delta^{2}-b^{2}\left(t_{k+1}^{1}-t_{k}^{1}\right)-\underset{l\leq k}{\sum }\int_{t_{k}^{2}}^{t_{k+1}^{2}}h^{22}\left(s-t_{l}^{2}\right)\;ds\\ & \;+\underset{l}{\sum }\int_{t_{k}^{2}}^{t_{k+1}^{2}}h^{12}\left(s-t_{l}^{1}\right)\;ds1_{\left\{t_{l}^{1}<t_{k}^{2}\right\}} \end{array}$$

and can be written in inner product form as

(32) $$\begin{array}{c} \langle u,\phi_{k}^{j}\rangle =q_{k}^{j}, \end{array}$$

with *q*_*k*_^*j*^ the right-hand side of (31) and *ϕ*_*k*_^*j*^ = 1_[*t*_*k*_^*j*^,*t*_*k*+1_^*j*^]_, for all *k*, *k* = 1, 2,…, *n*_*j*_ − 1, and *j*, *j* = 1, 2. With the *t*-transform in inner product form, the optimal consistent reconstruction is given by Theorem 12.

A simple example consisting of two symmetric neurons with parameters *δ* ^1^ = −*δ* ^2^ = *δ*, *κ* ^1^ = *κ* ^2^ = *κ*, and *b* ^1^ = −*b* ^2^ = *b* is considered here. The cross-feedback is of the form

(33) $$\begin{array}{c} h^{12}\left(t\right)=h^{21}\left(t\right)=c\;\exp\;\left(-\alpha t\right)\left(\frac{{\left(\alpha t\right)}^{5}}{5!}-\frac{{\left(\alpha t\right)}^{7}}{7!}\right)1_{\left[t\geq 0\right]}. \end{array}$$

No other feedback is present, that is, *h* ^11^ = *h* ^22^ = 0. The neuron parameters are *δ* = 0.75, *κ* = 0.01, and *b* = 3. In addition, *α* = 1/0.015 sec^−1^ and *c* = 1/3. Note that the impulse response of the filter has mean value zero. If the mean value is nonzero, the spike density of the ideal IAF neurons can be driven to zero or infinity.

The input was chosen to be the temporal contrast of an artificial (positive) input photocurrent. With *v* denoting the input photocurrent, the temporal contrast *u* is defined as

(34) $$\begin{array}{c} u\left(t\right)=\frac{d\log\;\left(v\left(t\right)\right)}{dt}=\frac{1}{v\left(t\right)}\frac{dv}{dt}. \end{array}$$

**Figure 3:**
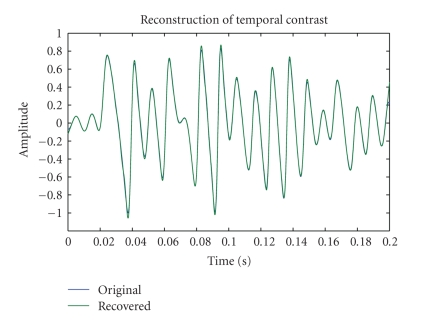
Recovery of temporal contrast from an ON-OFF IAF neural pair.

Clearly, even when the input bandwidth of the photocurrent *v* is known, the effective bandwidth of the actual neuron input *u* cannot be analytically estimated. The input photocurrent was bandlimited with bandwidth 100 Hz and had duration 200 milliseconds. Each neuron generated 75 spikes. The result of the recovery is shown in Figure 3. The SNR is equal to 28.75 dB.

## 4. Multi-Input Multi-Output Encoding and Consistent Stimulus Recovery

**Figure 4:**
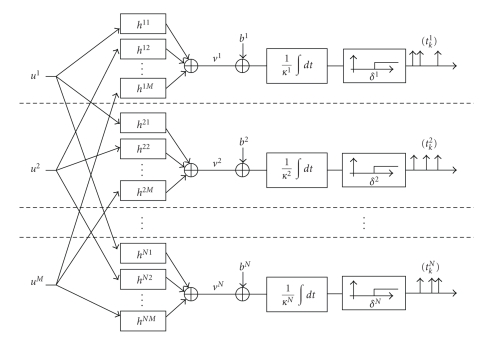
Multiple-Input Multiple-Output time encoding machine.

In this section we present our model of consistent information representation of *M*-dimensional vector signals using an *N* × *M*-dimensional filtering kernel and an ensemble of *N* integrate-and-fire neurons (see Figure 4). We assume without loss of generality that the neurons are ideal (nonleaky). Each component filter of the kernel receives input from one of the *M* component inputs, and its output is additively coupled into a single neuron. Finally, we describe an algorithm for stimulus reconstruction that is based on spline interpolation.

### 4.1. MIMO Model for Neural Encoding

Let *L*_*M*_^2^ = (*L* ^2^([0, *T*]))^*M*^ denote the space of *M*-dimensional, vector-valued functions of finite energy over the domain [0, *T*]. The general element of this space is **u** = [*u* ^1^, *u* ^2^,…, *u* ^*M*^]^*T*^, with *u* ^*i*^ = *u* ^*i*^(*t*), *t* ∈ [0, *T*], modeling the *i*th component of the input signal and *u* ^*i*^ ∈ *L* ^2^([0, *T*]) for all *i*, *i* = 1, 2,…, *M*. The space *L*_*M*_^2^ endowed with the inner product and norm defined by

(35) $$\begin{array}{ll} \langle u,v\rangle_{L_{M}^{2}} & =\overset{M}{\underset{i=1}{\sum }}\langle u^{i},v^{i}\rangle ,\\ {\left\|u\right\|}_{L_{M}^{2}}^{2} & =\overset{M}{\underset{i=1}{\sum }}{\left\|u^{i}\right\|}^{2}, \end{array}$$

respectively, is a Hilbert space. Let **H** : ℝ ↦ ℝ^*N*×*M*^ be a filtering kernel defined as

(36) $$\begin{array}{c} H\left(t\right)=\left[\begin{array}{cccc} h^{11}\left(t\right) & h^{12}\left(t\right) & \cdots & h^{1M}\left(t\right)\\ h^{21}\left(t\right) & h^{22}\left(t\right) & \cdots & h^{2M}\left(t\right)\\ \vdots & \vdots & \ddots & \vdots \\ h^{N1}\left(t\right) & h^{N2}\left(t\right) & \cdots & h^{NM}\left(t\right) \end{array}\right]. \end{array}$$

Filtering the signal **u** with the kernel **H** leads to an *N*-dimensional vector valued signal **v** defined by

(37) $$\begin{array}{c} v≜H*u=\left[\begin{array}{c} h^{11}*u^{1}+h^{12}*u^{2}+\cdots +h^{1M}*u^{M}\\ h^{21}*u^{1}+h^{22}*u^{2}+\cdots +h^{2M}*u^{M}\\ \vdots \\ h^{N1}*u^{1}+h^{N2}*u^{2}+\cdots +h^{NM}*u^{M} \end{array}\right]. \end{array}$$

Equation (37) can also be written in vector notation as

(38) $$\begin{array}{c} v^{j}={\left(h^{j}\right)}^{T}*u,\;\;\;j=1, 2,\ldots ,N, \end{array}$$

where **h** ^*j*^ = [*h* ^*j*1^, *h* ^*j*2^,…, *h* ^*jM*^]^*T*^ is the filtering vector of the neuron *j*, *j* = 1, 2,…, *N*. A bias *b* ^*j*^ is added to the component *v* ^*j*^ of the signal **v**, and the sum is passed through an integrate-and-fire neuron with integration constant (capacitance) *κ* ^*j*^ and threshold *δ* ^*j*^, for all *j*, *j* = 1, 2,…, *N* (see Figure 4). For simplicity we assume that the IAF neurons are ideal, that is, *R* ^*j*^ → ∞. Let *t*_*k*_^*j*^ denote the *k*th spike of the neuron *j*, with *k* = 1, 2,…, *n*_*j*_, where *n*_*j*_ is the number of spikes generated by neuron *j*, *j* = 1, 2,…, *N*. The Time Encoding Machine in Figure 4 maps, therefore, the input vector **u** into the vector time sequence (*t*_*k*_^*j*^), *j* = 1, 2,…, *N*, *k* = 1, 2,…, *n*_*j*_.

 The *t*-transform for the *j*th neuron can be written as

(39) $$\begin{array}{c} \int_{t_{k}^{j}}^{t_{k+1}^{j}}\left(v^{j}\left(s\right)+b^{j}\right)\;ds=\kappa^{j}\delta^{j}, \end{array}$$

or

(40) $$\begin{array}{c} \overset{M}{\underset{i=1}{\sum }}\int_{t_{k}^{j}}^{t_{k+1}^{j}}\left(h^{ji}*u^{i}\right)\left(s\right)\;ds=q_{k}^{j}, \end{array}$$

where *q*_*k*_^*j*^ = *κ* ^*j*^ *δ* ^*j*^ − *b* ^*j*^(*t*_*k* + 1_^*j*^ − *t*_*k*_^*j*^), for all *k*, *k* = 1, 2,…, *n*_*j*_ − 1, and all *j*, *j* = 1, 2,…, *N*. Note that, without any loss of generality, after firing all neurons are reset to the zero state. The *t*-transform (40) can be written in an inner product form as

(41) $$\begin{array}{c} \langle u,\phi_{k}^{j}\rangle =q_{k}^{j}, \end{array}$$

where

(42) $$\begin{array}{c} \phi_{k}^{j}={\tilde{h}}^{j}*1_{\left[t_{k}^{j},t_{k+1}^{j}\right]}=\left[\begin{array}{c} {\tilde{h}}^{j1}\\ {\tilde{h}}^{j2}\\ \vdots \\ {\tilde{h}}^{jM} \end{array}\right]*1_{\left[t_{k}^{j},t_{k+1}^{j}\right]} \end{array}$$

for all *j* = 1, 2,…, *N*, *k* = 1, 2,…, *n*_*j*_ − 1, and $\tilde{h}$ denotes the involution (time reversal) of *h*, that is, $\tilde{h}(t)=h(-t)$. Note that the impulse response of the filtering kernel **H** is not restricted to the interval [0, *T*] and can possibly have infinite support.

**Remark 13**

An implicit assumption in writing the *t*-transform in the inner product form (41) is that the sampling functions *ϕ*_*k*_^*j*^ belong to *L*_*M*_^2^. A sufficient condition for the latter is that all filters are bounded-input bounded-output (BIBO) stable, that is, ∫_ℝ_ |*h* ^*ji*^(*s*)| *ds* < ∞ for all *i*, *i* = 1, 2,…, *M*, and all *j*, *j* = 1, 2,…, *N*.

### 4.2. Consistent Stimulus Recovery

The optimal consistent reconstruction is given by the solution of the following spline interpolation problem:

(43) $$\begin{array}{c} \hat{u}=\underset{\langle u,\phi_{k}^{j}\rangle =q_{k}^{j}}{\text{argmin}}{\left(\overset{M}{\underset{i=1}{\sum }}{\left\|\frac{d^{2}u^{i}}{{dt}^{2}}\right\|}^{2}\right)}^{1/2}. \end{array}$$

We have the following result.

**Theorem 14**

*Assume that at time 0 the membrane potential of all neurons is at the rest value 0. The optimal consistent reconstruction* $\hat{u}$ *is unique and can be written as*

(44) $$\begin{array}{c} \hat{u}\left(t\right)=d_{0}+d_{1}t+\overset{N}{\underset{j=1}{\sum }}\overset{n_{j}-1}{\underset{k=1}{\sum }}c_{k}^{j}\psi_{k}^{j}\left(t\right), \end{array}$$

*where* **d**_0_, **d**_1_ ∈ ℝ^*M*^ *and*

(45) $$\begin{array}{ll} \psi_{k}^{j}\left(t\right) & =\left(\left[\begin{array}{c} {\tilde{h}}^{j1}\\ {\tilde{h}}^{j2}\\ \vdots \\ {\tilde{h}}^{jM} \end{array}\right]*1_{\left[t_{k}^{j},t_{k+1}^{j}\right]}*{\left|\cdot \right|}^{3}\right)\left(t\right)\\ & \begin{array}{c} =\left[\begin{array}{c} \int_{t_{k}^{j}}^{t_{k+1}^{j}}\left({\tilde{h}}^{j1}\left(\cdot \right)*{\left|\cdot \right|}^{3}\right)\left(t-s\right)\;ds\\ \int_{t_{k}^{j}}^{t_{k+1}^{j}}\left({\tilde{h}}^{j2}\left(\cdot \right)*{\left|\cdot \right|}^{3}\right)\left(t-s\right)\;ds\\ \vdots \\ \int_{t_{k}^{j}}^{t_{k+1}^{j}}\left({\tilde{h}}^{jM}\left(\cdot \right)*{\left|\cdot \right|}^{3}\right)\left(t-s\right)\;ds \end{array}\right]. \end{array} \end{array}$$

*With* **p** = [**p** ^1^; **p** ^2^; … ; **p** ^*N*^]^*T*^, **p** ^*j*^ ∈ ℝ^(*n*_*j*_−1)×*M*^, *and* **r** *similarly defined, the reconstruction coefficients are given in matrix form by*

(46) $$\left[\begin{array}{c} c\\ d_{0}\\ d_{1} \end{array}\right]={\left[\begin{array}{ccc} G & p & r\\ p^{T} & 0 & 0\\ r^{T} & 0 & 0 \end{array}\right]}^{+}\cdot \left[\begin{array}{c} q\\ 0\\ 0 \end{array}\right],$$

*with*

(47) $$\begin{array}{cc} {\left[p^{j}\right]}_{ki} & =\langle 1,\phi_{k}^{ji}\rangle ,\\ {\left[r^{j}\right]}_{ki} & =\langle 1,\phi_{k}^{ji}\rangle ,\\ {\left[G^{ij}\right]}_{kl} & =\langle \phi_{k}^{i},\psi_{l}^{j}\rangle , \end{array}$$

*where the inner products are restricted to the interval* [0, *T*].

**Proof**

The proof is based on Theorem 4.

**Remark 15**

Note that since the signal reconstruction is set up as a spline interpolation problem, the algorithm presented in Theorem 14 above will produce a solution that depends on both the filtering kernel **H** and the spiking mechanism of the population of neurons. We will briefly mention here conditions of no information loss due to filtering. If *ℱ* denotes the Fourier transform, we have

(48) $$\begin{array}{c} \left(ℱv\right)\left(\omega \right)=\left(ℱH\right)\left(\omega \right)\cdot \left(ℱu\right)\left(\omega \right). \end{array}$$

The requirement for no information loss implies that *ℱ* **H**, the filtering kernel in the Fourier domain, has rank *M* for all frequencies of interest (here for all *ω* ∈ ℝ). A trivial necessary condition that comes out of the rank condition is that *N* ≥ *M*; that is, the number of neurons that encode the stimulus must be at least equal to the number of its components. This intuitive argument has important ramifications in experimental neuroscience as it shows that, in general, multivariate stimuli (e.g., video sequences) cannot be efficiently represented by the spike train of a single neuron or a small neural population. Rather, the spike trains from a larger population of neurons that encode the same stimulus needs to be used.

### 4.3. Example: Delay Filter Bank

We present the realization of the recovery algorithm for a filtering kernel that induces * arbitrary, but known, delays, and weights* on the stimulus. The kernel models dendritic tree latencies in sensory neurons (motor, olfactory) [8] or, in general, delays and synaptic weights between groups of pre- and postsynaptic neurons. In order to incorporate these delays, we assume that the stimuli are defined on a time window larger than [0, *T*]. The inner product, however, is restricted to the time interval [0, *T*].

Each filter *h* ^*ji*^ delays the stimulus in time by an arbitrary positive amount *α* ^*ji*^ and scales it by an arbitrary real number *w* ^*ji*^, for all *j* = 1, 2 …, *N*, and all *i* = 1, 2,…, *M*. Consequently, *h* ^*ji*^ = *w* ^*ji*^ *δ*(*t* − *α* ^*ji*^) and ${\tilde{h}}^{ji}=w^{ji}\delta (t+\alpha^{ji})$. From now on let *τ*_*k*_^*ji*^ = *t*_*k*_^*j*^ + *α* ^*ji*^ for all *i*, *i* = 1, 2 …, *M* all *j*, *j* = 1, 2,…, *N* and all *k*, *k* = 1, 2,…, *n*_*j*_ − 1.

The representation functions *ψ*_*k*_^*j*^, *j* = 1, 2,…, *N*, *k* = 1, 2,…, *n*_*j*_ − 1, of (45) are given by

(49) $$\begin{array}{c} \psi_{k}^{j}\left(t\right)=\left[\begin{array}{c} w^{j1}\int_{\tau_{k}^{j1}}^{\tau_{k+1}^{j1}}{\left|t-s\right|}^{3}\;ds\\ w^{j2}\int_{\tau_{k}^{j2}}^{\tau_{k+1}^{j2}}{\left|t-s\right|}^{3}\;ds\\ \vdots \\ w^{jM}\int_{\tau_{k}^{jM}}^{\tau_{k+1}^{jM}}{\left|t-s\right|}^{3}\;ds \end{array}\right] \end{array}$$

for all *t* ∈ [0, *T*]. Note that the general term of (49) can be expressed analytically similarly to (19).

 The entries of (46) can be computed from (47) as

(50) $$\begin{array}{ll} {\left[p^{j}\right]}_{ki} & =\{\begin{array}{cc} w^{ji}\left(\tau_{k+1}^{ji}-\tau_{k}^{ji}\right), & \tau_{k+1}^{j}<T,\\ w^{ji}\left(T-\tau_{k}^{ji}\right), & \tau_{k}^{ji}<T<\tau_{k+1}^{ji},\\ 0, & T<\tau_{k}^{ji}, \end{array}\\ {\left[r^{j}\right]}_{ki} & =\{\begin{array}{cc} 0.5w^{ji}\left({\left(\tau_{k+1}^{ji}\right)}^{2}-{\left(\tau_{k}^{ji}\right)}^{2}\right), & \tau_{k+1}^{j}<T,\\ 0.5w^{ji}\left(T^{2}-{\left(\tau_{k}^{ji}\right)}^{2}\right), & \tau_{k}^{ji}<T<\tau_{k+1}^{ji},\\ 0, & T<\tau_{k}^{ji}, \end{array}\\ {\left[G^{ij}\right]}_{kl} & =\langle \phi_{k}^{i},\psi_{l}^{j}\rangle =\overset{M}{\underset{m=1}{\sum }}\langle \phi_{k}^{im},\psi_{l}^{jm}\rangle \\ & =\overset{M}{\underset{m=1}{\sum }}w^{im}w^{jm}\int_{\tau_{k}^{im}}^{\tau_{k+1}^{im}}\psi_{l}^{jm}\left(t\right)\;dt‍. \end{array}$$

Note that the entries of **G** can be computed analytically as

(51) $$\begin{array}{ll} & {\left[G^{ij}\right]}_{kl}\\ & =\overset{M}{\underset{m=1}{\sum }}\frac{w^{im}w^{jm}}{20}\\ & \;\times [({\left(\tau_{k+1}^{im}-\tau_{l}^{jm}\right)}^{5}+{\left(\tau_{k}^{im}-\tau_{l+1}^{jm}\right)}^{5}\\ & \;\;\;\;-{\left(\tau_{k}^{im}-\tau_{l}^{jm}\right)}^{5}-{\left(\tau_{k+1}^{im}-\tau_{l+1}^{jm}\right)}^{5})\cdot 1\left(\tau_{l+1}^{jm}\leq \tau_{k}^{im}\right)\\ & \;\;\;+({\left(\tau_{k+1}^{im}-\tau_{l}^{jm}\right)}^{5}-{\left(\tau_{k}^{im}-\tau_{l+1}^{jm}\right)}^{5}-{\left(\tau_{k}^{im}-\tau_{l}^{jm}\right)}^{5}\\ & \;\;\;\;-{\left(\tau_{k+1}^{im}-\tau_{l+1}^{jm}\right)}^{5})\cdot 1\left(\tau_{l}^{jm}\leq \tau_{k}^{im}\leq \tau_{l+1}^{jm}\leq \tau_{k+1}^{im}\right)\\ & \;\;\;+({\left(\tau_{k+1}^{im}-\tau_{l}^{jm}\right)}^{5}-{\left(\tau_{k}^{im}-\tau_{l+1}^{jm}\right)}^{5}-{\left(\tau_{k}^{im}-\tau_{l}^{jm}\right)}^{5}\\ & \;\;\;\;+{\left(\tau_{k+1}^{im}-\tau_{l+1}^{jm}\right)}^{5})\cdot 1\left(\tau_{l}^{jm}\leq \tau_{k}^{im}\leq \tau_{k+1}^{im}\leq \tau_{l+1}^{jm}\right)\\ & \;\;\;+({\left(\tau_{k+1}^{im}-\tau_{l}^{jm}\right)}^{5}-{\left(\tau_{k}^{im}-\tau_{l+1}^{jm}\right)}^{5}+{\left(\tau_{k}^{im}-\tau_{l}^{jm}\right)}^{5}\\ & \;\;\;\;-{\left(\tau_{k+1}^{im}-\tau_{l+1}^{jm}\right)}^{5})\cdot 1\left(\tau_{k}^{im}\leq \tau_{l}^{jm}\leq \tau_{l+1}^{jm}\leq \tau_{k+1}^{im}\right)\\ & \;\;\;+({\left(\tau_{k+1}^{im}-\tau_{l}^{jm}\right)}^{5}-{\left(\tau_{k}^{im}-\tau_{l+1}^{jm}\right)}^{5}+{\left(\tau_{k}^{im}-\tau_{l}^{jm}\right)}^{5}\\ & \;\;\;\;+{\left(\tau_{k+1}^{im}-\tau_{l+1}^{jm}\right)}^{5})\cdot 1\left(\tau_{k}^{im}\leq \tau_{l}^{jm}\leq \tau_{k+1}^{im}\leq \tau_{l+1}^{jm}\right)\\ & \;\;\;+(-{\left(\tau_{k+1}^{im}-\tau_{l}^{jm}\right)}^{5}-{\left(\tau_{k}^{im}-\tau_{l+1}^{jm}\right)}^{5}+{\left(\tau_{k}^{im}-\tau_{l}^{jm}\right)}^{5}\\ & \;\;\;\;+{\left(\tau_{k+1}^{im}-\tau_{l+1}^{jm}\right)}^{5})\cdot 1\left(\tau_{k+1}^{im}\leq \tau_{l}^{jm}\right)]. \end{array}$$

The vector-valued signal **u**(*t*) = [*u* ^1^(*t*), *u* ^2^(*t*), *u* ^3^(*t*)]^*T*^ has three bandlimited components (*M* = 3) each with the same bandwidth Ω = 2*π* · 100 Hz and time length *T* = 100 millisecond. In total, 9 IAF neurons were used to recover the signal (*N* = 9). The delays were drawn randomly from an exponential distribution with mean 3 millisecond. The biases *b* ^*j*^ and thresholds *δ* ^*j*^, *j* = 1, 2 …, 9, were drawn from uniform distributions on the intervals [2.3, 3.3] and [0.5, 1.5], respectively. Finally, *κ* ^*j*^ = 0.01 for all *j* = 1, 2,…, 9.

**Figure 5:**
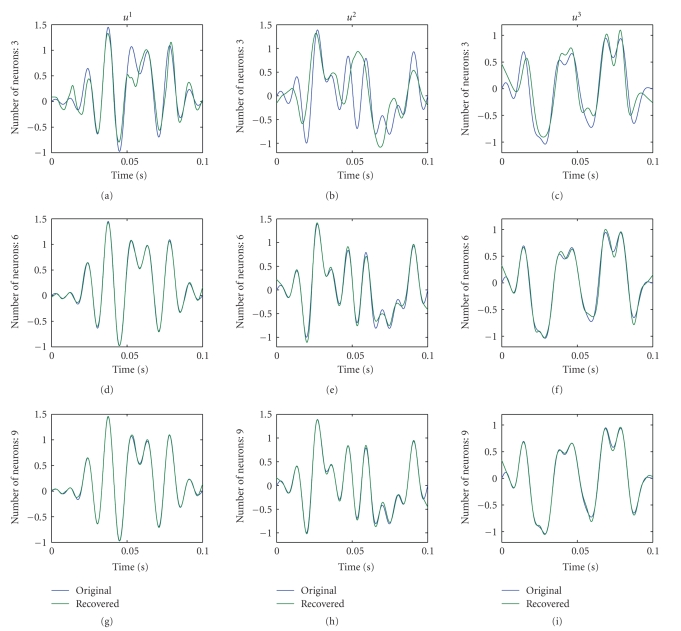
Recovery of the 3-dimensional input vector valued signal. In each row the original (blue) and recovered (green) signals are shown for the indicated number of neurons used for recovery. The columns correspond to each component of the input signal.

The recovered stimuli using the spikes from 3, 6, and 9 neurons, respectively, are depicted from top to bottom in Figure 5. For each component, the recovered signal converges to the original one.

**Figure 6:**
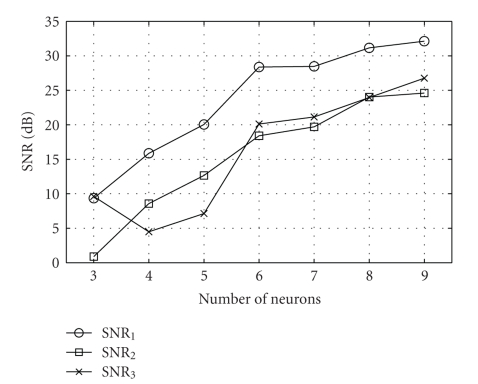
SNR as a function of the number of neurons.

Figure 6 shows the SNR corresponding to the recovery of each stimulus component when 3,4,…, 9 neurons are used. Figure 6 demonstrates that overall, as more neurons are added to the representation of the stimulus, the SNR of all stimulus components increases. An exception is observed in the recovery of the component *u* ^3^; the addition of a neuron from three to four leads to a decrease of the SNR. Note, however, that the SNR for the recovery of the entire vector-valued stimulus **u** increases with the addition of the fourth neuron from 7.71 dB to 12.23 dB (data not shown).

## 5. Discussion

The methodology of interpolating splines presented here applies to the deterministic case where the input stimulus and the LIF neurons have low noise levels. It ties in naturally with theoretical results that show that neural encoding of bandlimited signals leads to perfect signal reconstruction if Nyquist-type rate conditions are satisfied [5].

In neuromorphic engineering applications the noise levels can be kept low. Neuronal spike trains, however, often exhibit strong variability in response to identical inputs due to various noise sources. For stimuli encoded with neural circuits the problem of optimal reconstruction can be formulated as a smoothing spline problem [4]. This case is presented analytically in [7] for a slightly less general setup, where the signals belong to a Reproducing Kernel Hilbert Space [19]. A reconstruction of stimuli encoded with LIF neurons using both smoothing and interpolating splines offers an additional alternative. Thus, the methodology of spline theory provides a general framework for the optimal reconstruction of signals on a finite time horizon.

The methodology presented here can be applied to the reconstruction of stimuli encoded with neurons that belong to other model classes of interest. An example is provided by neuron models with level crossing spiking mechanisms and feedback that have been investigated in [16]. More generally, the *t*-transform of any neuron model with piecewise linear dynamics can be described by a set of linear projections. Neurons with linear dynamics have been shown to express complex spiking behaviors [20–22].

The MIMO architecture presented here consists of a linear, time-invariant filtering kernel that is separated from the neural spiking mechanism. By relaxing the time-invariance property and embedding spike-triggered reseting mechanisms at the level of the filtering kernel, more complex transformations can be modeled. Consequently dendritic trees incorporating compartmental neuron models and spike backpropagation [23] can be analyzed with the methodology advanced in this paper. The aforementioned architectures will be the subject of future research.

## Appendices

### A. Interpolation Splines in Hilbert Spaces

We assume throughout that stimuli *u* belong to the space of functions of finite energy over a domain *𝒯*, that is, *u* ∈ *L* ^2^(*𝒯*). The information available to a decoder is a set of measurements

(A.1) $$\begin{array}{c} \langle \phi_{k},u\rangle =q_{k}, \end{array}$$

where *ϕ*_*k*_ ∈ *L* ^2^(*𝒯*) are known functions and *k* = 1, 2,…, *n*. The inner products can be written in operator form as

(A.2) $$\begin{array}{c} Lu=q, \end{array}$$

where **q** = [*q*_1_, *q*_2_,…, *q*_*n*_]^*T*^, and **L** : *L* ^2^(*𝒯*) ↦ ℝ^*n*^ is a linear operator defined by

(A.3) $$Lu={\left[\langle \phi_{1},u\rangle ,\langle \phi_{2},u\rangle ,\ldots ,\langle \phi_{n},u\rangle \right]}^{T}.$$

Finding *u* by inverting (A.2) is, in general, an ill-posed problem. Additional “smoothness” conditions are needed. We introduce these by requiring that the reconstructed signal minimizes a quadratic criterion ||*Ju*||, where *J* : *L* ^2^(*𝒯*) ↦ *Y* is a bounded linear operator, *Y* is the range of *J*, and || · || denotes the standard *L* ^2^-norm over *𝒯*.

Definition 1 The solution to the interpolation problem

(A.4) $$\begin{array}{c} \hat{u}=\underset{u:Lu=q}{\text{argmin}}{\left\|Ju\right\|}^{2} \end{array}$$

is called an interpolation spline corresponding to the initial data **q**, the measurement operator **L**, and the energy operator *J*.

We restrict ourselves to the case where the operator *J* has a finite dimensional kernel of dimension *m*. The following standard theorem establishes necessary conditions for the existence and uniqueness of the interpolation spline. For a proof see [13].

Theorem 2
*If ker*(*L*) ∩ ker(*J*) = {0}, *and the range of *J* is closed, then there exists a unique interpolation spline corresponding to the data* **q**, *the measurement operator* **L**, *and the energy operator *J**.

In order to derive the general form of the interpolation spline, we introduce the notion of reproducing kernel for a Hilbert space with respect to the energy operator *J*. This notion generalizes reproducing kernels associated with Reproducing Kernel Hilbert Spaces [19].

Definition 3 The function *K* : *𝒯* × *𝒯* ↦ ℝ is called the reproducing kernel of the space *L* ^2^(*𝒯*) with respect to the energy operator *J*, if

1. for any functional *L* : *L* ^2^(*𝒯*) ↦ ℝ, the function *f*(*s*) = *LK*(*s*, ·) lies in *L* ^2^(*𝒯*);
2. any functional *L* : *L* ^2^(*𝒯*) ↦ ℝ that vanishes on the kernel of *J*, that is,

(A.5) $$\begin{array}{c} Lu=0,\;\;\;\forall u\in \text{ker}\left(J\right) \end{array}$$

can be represented as

(A.6) $$\begin{array}{c} Lu=B\left(LK\left(s,\cdot \right),u\right),\;\;\;\forall u\in L^{2}\left(𝒯\right). \end{array}$$

Here *B* : *L* ^2^(*𝒯*) × *L* ^2^(*𝒯*) ↦ ℝ is a bilinear form defined by

(A.7) $$\begin{array}{c} B\left(u,v\right)=\frac{1}{4}\left({\left\|J(u+v)\right\|}^{2}-{\left\|J(u-v)\right\|}^{2}\right). \end{array}$$

Theorem 4 
*The solution to the spline interpolation problem is given by*

(A.8) $$\begin{array}{c} \hat{u}=\overset{m}{\underset{i=1}{\sum }}d_{i}\chi_{i}+\overset{n}{\underset{k=1}{\sum }}c_{k}\psi_{k}, \end{array}$$

*where the set of functions* (*χ*_*i*_), *i* = 1, 2,…, *m*, *forms an orthogonal basis for* ker(*J*) *and*

(A.9) $$\begin{array}{c} \psi_{k}\left(s\right)=\langle \phi_{k},K\left(s,\cdot \right)\rangle . \end{array}$$

*With* **d** = [*d*_1_, *d*_2_,…, *d*_*m*_]^*T*^, **c** = [*c*_1_, *c*_2_,…, *c*_*n*_]^*T*^, and **q** = [*q*_1_, *q*_2_,…, *q*_*n*_]^*T*^, *the coefficients* **c** *and* **d** *satisfy the matrix equations*

(A.10) $$\left[\begin{array}{cc} G & F\\ F^{T} & 0 \end{array}\right]\left[\begin{array}{c} c\\ d \end{array}\right]=\left[\begin{array}{c} q\\ 0 \end{array}\right].$$

*Moreover* **G** *is an* *n* × *n* *matrix and* **F** *is an* *n* × *m* *matrix with entries given by*

(A.11) $$\begin{array}{l} {\left[G\right]}_{kl}=\langle \phi_{k},\psi_{l}\rangle ,\\ {\left[F\right]}_{kl}=\langle \phi_{k},\chi_{l}\rangle , \end{array}$$

*for all* *i* = 1, 2,…, *n*, *j* = 1, 2,…, *n* *and all* *l* = 1, 2,…, *m*.

Proof For the representation result of (A.8) see [13]. By substituting (A.8) into (A.2), we obtain

(A.12) $$\begin{array}{c} \left[\begin{array}{cc} G & F \end{array}\right]\cdot \left[\begin{array}{c} c\\ d \end{array}\right]=q. \end{array}$$

For the rest of the equations define

(A.13) $$\begin{array}{c} J_{*}\left(u,c\right)={\left\|Ju\right\|}^{2}-2\overset{n}{\underset{k=1}{\sum }}c_{k}\left(\langle \phi_{k},u\rangle -q_{k}\right), \end{array}$$

where the entries of the vector **c** are the Lagrange multipliers. If $\hat{u}$ is the optimal consistent reconstruction and *v* ∈ *L* ^2^(*𝒯*), then

(A.14) $$\begin{array}{c} {\frac{\partial }{\partial \alpha }J_{*}\left(\hat{u}+\alpha v,c\right)|}_{\alpha =0}=0\Rightarrow B\left(\hat{u},v\right)=\overset{n}{\underset{k=1}{\sum }}c_{k}\langle \phi_{k},v\rangle . \end{array}$$

From the Cauchy-Schwarz inequality with *v* ∈ker(*J*), we have

(A.15) $$\begin{array}{c} B\left(u,v\right)=0. \end{array}$$

Therefore, for each of the basis functions of ker(*J*), *χ*_1_,…, *χ*_*m*_,

(A.16) $$\begin{array}{c} \overset{n}{\underset{k=1}{\sum }}c_{k}\langle \phi_{k},\chi_{j}\rangle =0, \end{array}$$

which in matrix form can be written as

(A.17) $$F^{T}c=0,$$

with **F** defined as in (A.11). Combining (A.12) with (A.17) we obtain (A.10). For more information see [13].

### B. Reproducing Kernels for MIMO Signal Reconstruction

Let *L*_*M*_^2^ = (*L* ^2^(*𝒯*))^*M*^ be the space of *M*-dimensional vector-valued functions defined over the domain *𝒯*. The space equipped with inner product and norm given by (35) is a Hilbert space. Suppose that we seek a consistent reconstruction that also minimizes the energy operator

(B.1) $$\begin{array}{c} J_{M}\left(u\right)= \end{array}{\left(\overset{M}{\underset{i=1}{\sum }}{\left\|\frac{d^{m}u^{i}}{{dt}^{m}}\right\|}^{2}\right)}^{1/2}.$$

Lemma 5
*The reproducing kernel for the Hilbert space* *L*_*M*_^2^ *with respect to the energy operator* *J*_*M*_ *is given by*

(B.2) $$\begin{array}{c} K\left(t,s\right)=\frac{{\left(-1\right)}^{m}}{2\left(2m-1\right)!}{\left|t-s\right|}^{2m-1}\cdot {\left[1,1,\ldots ,1\right]}^{T}. \end{array}$$

Proof It can be shown that the reproducing kernel can be written in the form

(B.3) $$\begin{array}{c} K\left(t,s\right)=E\left(t-s\right), \end{array}$$

where **E**(·) is the fundamental solution for the *m*th-iterated Laplacian

(B.4) $$\begin{array}{c} \Delta^{m}E=\delta . \end{array}$$

For univariate functions the iterated Laplacian is equal to the 2*m*th order derivative of each component and the result follows. A complete proof can be found in a general setting in [24].

Note that for *m* = 2 the kernel becomes

(B.5) $$K\left(t,s\right)=\frac{1}{12}{\left|t-s\right|}^{3}\cdot {\left[1,1,\ldots ,1\right]}^{T}.$$

For the general representation result (A.8), the scalar factor can be absorbed into the coefficients.

## References

[1] Lazar AA (2004). Time encoding with an integrate-and-fire neuron with a refractory period. *Neurocomputing*.

[2] Lazar AA, Tóth LT (2004). Perfect recovery and sensitivity analysis of time encoded bandlimited signals. *IEEE Transactions on Circuits and Systems I*.

[3] Christensen O (2003). *An Introduction to Frames and Riesz Bases*.

[4] Wahba G (1990). *Spline Models for Observational Data*.

[5] Lazar AA, Pnevmatikakis EA (2008). Faithful representation of stimuli with a population of integrate-and-fire neurons. *Neural Computation*.

[6] Lazar AA, Pnevmatikakis EA A video time encoding machine.

[7] Lazar AA, Pnevmatikakis EA (2009). Reconstruction of sensory stimuli encoded with integrate-and-fire neurons with random thresholds. *EURASIP Journal on Advances in Signal Processing*.

[8] Fain GL (2003). *Sensory Transduction*.

[9] Song D, Chan RH, Marmarelis VZ, Hampson RE, Deadwyler SA, Berger TW (2007). Nonlinear dynamic modeling of spike train transformations for hippocampal-cortical prostheses. *IEEE Transactions on Biomedical Engineering*.

[10] Zanos TP, Courellis SH, Berger TW, Hampson RE, Deadwyler SA, Marmarelis VZ (2008). Nonlinear modeling of causal interrelationships in neuronal ensembles. *IEEE Transactions on Neural Systems and Rehabilitation Engineering*.

[11] Kim S-P, Sanchez JC, Rao YN (2006). A comparison of optimal MIMO linear and nonlinear models for brain-machine interfaces. *Journal of Neural Engineering*.

[12] Serrano-Gotarredona R, Serrano-Gotarredona T, Acosta-Jiménez A (2008). On real-time AER 2-D convolutions hardware for neuromorphic spike-based cortical processing. *IEEE Transactions on Neural Networks*.

[13] Bezhaev AI, Vasilenko VA (2001). *Variational Theory of Splines*.

[14] Bartels RH, Beatty JC, Barsky BA (1987). *An Introduction to Splines for Use in Computer Graphics & Geometric Modeling*.

[15] Unser M, Aldroubi A, Eden M (1993). B-spline signal processing—part I: theory. *IEEE Transactions on Signal Processing*.

[16] Lazar AA, Pnevmatikakis EA Consistent recovery of stimuli encoded with a neural ensemble.

[17] Kybic J, Blu T, Unser M (2002). Generalized sampling: a variational approach. I: theory. *IEEE Transactions on Signal Processing*.

[18] Masland RH (2001). The fundamental plan of the retina. *Nature Neuroscience*.

[19] Berlinet A, Thomas-Agnan C (2004). *Reproducing Kernel Hilbert Spaces in Probability and Statistics*.

[20] Izhikevich EM (2001). Resonate-and-fire neurons. *Neural Networks*.

[21] Mihalaş Ş, Niebur E (2009). A generalized linear integrate-and-fire neural model produces diverse spiking behaviors. *Neural Computation*.

[22] Jolivet R, Kobayashi R, Rauch A, Naud R, Shinomoto S, Gerstner W (2008). A benchmark test for a quantitative assessment of simple neuron models. *Journal of Neuroscience Methods*.

[23] Bressloff PC, Taylor JG (1994). Dynamics of compartmental model neurons. *Neural Networks*.

[24] Duchon J, Schempp W, Zeller K (1977). Splines minimizing rotation-invariant semi-norms in sobolev spaces. *Constructive Theory of Functions of Several Variables*.

